# Berberine elevates cardiolipin in heart of offspring from mouse dams with high fat diet-induced gestational diabetes mellitus

**DOI:** 10.1038/s41598-021-95353-4

**Published:** 2021-08-04

**Authors:** Laura K. Cole, Genevieve C. Sparagna, Marilyne Vandel, Bo Xiang, Vernon W. Dolinsky, Grant M. Hatch

**Affiliations:** 1 Diabetes Research Envisioned and Accomplished in Manitoba (DREAM) Theme, Department of Pharmacology and Therapeutics, Children’s Hospital Research Institute of Manitoba, Rady Faculty of Health Sciences, University of Manitoba, 501C JBRC, 715 McDermot Avenue, Winnipeg, MB R3E 3P4 Canada; 2 Division of Cardiology, Department of Medicine, University of Colorado Anschutz Medical Center, Aurora, USA

**Keywords:** Biochemistry, Biological techniques, Cell biology, Molecular biology, Cardiology, Diseases, Medical research

## Abstract

Berberine (BBR) is an isoquinoline alkaloid from plants known to improve cardiac mitochondrial function in gestational diabetes mellitus (GDM) offspring but the mechanism is poorly understood. We examined the role of the mitochondrial phospholipid cardiolipin (CL) in mediating this cardiac improvement. C57BL/6 female mice were fed either a Lean-inducing low-fat diet or a GDM-inducing high-fat diet for 6 weeks prior to breeding. Lean and GDM-exposed male offspring were randomly assigned a low-fat, high-fat, or high-fat diet containing BBR at weaning for 12 weeks. The content of CL was elevated in the heart of GDM offspring fed a high fat diet containing BBR. The increase in total cardiac CL was due to significant increases in the most abundant and functionally important CL species, tetralinoleoyl-CL and this correlated with an increase in the expression of the CL remodeling enzyme tafazzin. Additionally, BBR treatment increased expression of cardiac enzymes involved in fatty acid uptake and oxidation and electron transport chain subunits in high fat diet fed GDM offspring. Thus, dietary BBR protection from cardiac dysfunction in GDM exposed offspring involves improvement in mitochondrial function mediated through increased synthesis of CL.

## Introduction

Maternal obesity or overweight before and throughout pregnancy is a major risk factor for development of gestational diabetes mellitus (GDM)^1,2^. Diagnosis of GDM has increased dramatically over the past two decades due in part to the obesity epidemic^3–5^. For example, 60% of all women are classified as being overweight at the beginning of pregnancy and approximately 30% of all women are classified as being obese at the beginning of pregnancy^6^. Maternal obesity and diabetes during pregnancy predispose offspring to the development of cardiometabolic diseases, including obesity and diabetes^7,8^. Moreover, we have shown that diet-induced GDM exposure accelerates postnatal high fat diet-induced obesity and cardiometabolic dysfunction in the offspring^12,14,15^. The neonatal period represents a key intervention window for reducing the lifetime disease risk of the effects of GDM^9^. Earlier interventions in vulnerable populations from GDM pregnancies could prevent the development of cardiometabolic disease, but presently few evidence-based strategies are known.

Berberine (BBR) is an isoquinoline alkaloid from plants that improves insulin sensitivity and reduces blood glucose levels^10,11^. The actions of BBR are also known to promote improved mitochondrial function^11^. We recently demonstrated that supplemental BBR in a high fat diet reduced adiposity and improved cardiac dysfunction in offspring of mouse dams with GDM^12^. In GDM offspring there were significant reductions in ejection fraction and fractional shortening. BBR improved the contractility of the left ventricle as reflected by a normalized myocardial performance index. In addition, BBR improved both diastolic and systolic function in GDM offspring. Thus, BBR treatment of high fat fed offspring improved overall metabolic health and cardiac mitochondrial function; however, the mechanism for the cardioprotective effect was unknown. The liver plays a key role in dietary lipid handling and metabolism. In addition, fatty acids derived from triacylglycerol are a major oxidative fuel for the heart and skeletal muscle. Optimal mitochondrial function in these tissues is paramount for maintaining healthy body weight, insulin sensitivity and cardiovascular function. Since cardiolipin (CL) is a key mitochondrial membrane phospholipid known to regulate cardiac mitochondrial bioenergetics^13^, we examined if changes in CL were, in part, responsible for the cardioprotective effects of BBR in the heart of high fat fed GDM offspring.

## Materials and methods

### Animal care and food preparation

The study was conducted with approval of the University of Manitoba Animal Policy and Welfare Committee. All methods were performed in accordance with the Canadian Council on Animal Care guidelines and regulations. This study is reported in accordance with ARRIVE guidelines. Animals were maintained in an environmentally controlled facility (12 h light/dark cycle) with ad libitium access to food and water. A diet-induced GDM rodent model used was previously described^12,14,15^. 6 week old female C57BL/6 mice were fed either a low-fat (10% kcal, Research Diets D12450B) or a high-fat/ sucrose diet (45% kcal, Research Diets D12451) for 6 weeks prior to mating for 4 days with 6–10 week old low-fat fed male C57BL/6 mouse. The diet assigned to the dams was continued during mating, pregnancy and suckling periods. Resulting litters were culled at a maximum of eight to prevent nutritional deprivation of offspring. Male offspring were weaned at 3 weeks and randomly assigned to one of three diets (1) low fat (10% kcal) (LF), (2) high-fat/sucrose (45% kcal) (HF), or (3) high fat/sucrose (45% kcal) diet supplemented with BBR (160 mg/kg/day) (HFB) for a 12 week period. Rodent food was prepared with BBR by generating agar blocks using powered rodent diet purchased commercially from Research Diets (Low fat—D12450B, or high fat D12451). Agar 14.5 g/kg (Sigma) and the indicated LF or HF diet 435 g/kg were dissolved in heated water (1 kg) with or without BBR 1.45 g/kg (Sigma). The mixture was cooled to 4 °C, cut into blocks and were placed in animal hoppers every 2–3 days^12^. All animals were 15 weeks of age when experiments were conducted. At 15 weeks of age, offspring underwent a 16 h fast before being anesthetized with 1–1.5% isoflurane and then euthanized by cervical dislocation.

### Blood measurements

Non-esterified fatty acids (NEFA), triglycerides (TG), ketones and alanine amino transferase (ALT) were determined after 12 weeks of experimental diet. Plasma was collected from blood in tubes coated with EDTA (Sarstedt, Numbrecht, Germany) followed by centrifugation at 6000 RPM at 4 °C for 20 min. Commercially available kits were used to measure plasma non-esterified fatty acids, ketones, triglyceride (Wako Diagnositic), and alanine aminotransferase (Biotron Diagnostic). All assays were analyzed using an Epoch Biotek microplate spectrophotometer (Agilent Technologies, ON, Canada).

### Tissue lipid measurements

A commercially available kit was used to measure tissue triglyceride (Wako Diagnositics). Phospholipids from tissue homogenates including phosphatidylethanolamine (PE), phosphatidylserine (PS), phosphatidylcholine (PC) and phosphatidylinositol (PI) were quantitated by lipid phosphorus assay^16^. Molecular species of CL were quantitated from tissue homogenates by HPLC coupled to electrospray ionization mass spectrometry^17^. The total CL was calculated from the sum of the eight most prominent CL species.

### RNA isolation and quantitative RT-PCR analysis

Total RNA was isolated from cardiac, skeletal muscle (soleus) or liver tissue using an RNeasy^®^ kit (Qiagen) and first-strand cDNA synthesis performed (2 µg total RNA) with SuperScript II (Invitrogen). PCR was performed using Eppendorf Realplex^2^ instrument with the gene specific primers (IDT) as indicated (Table 1). All mRNA levels were quantitated using a standard curve followed by normalized to the geometric mean (geomean) of Tata Box binding protein (TBP) and Transcription factor IIB (TFIIB)^18^.

### Statistical analysis

Data are expressed either as means ± standard error of the mean (SEM) or as means with individual data points representing each individual animal. Comparisons between Lean and GDM Dams were performed by unpaired two-tailed Student’s t-test. Comparisons between Lean and GDM exposed HF, HFB and LF fed offspring were determined separately for male and female animals using two-way analysis of variance using Tukey post-hoc analysis. When appropriate adjustments were made for multiple comparisons using Bonferroni correction. Analysis of covariance was used to determine whether body weight was a confounding factor within indirect calorimetry measurements. For each measurement, the offspring were derived from multiple litters. A probability value of < 0.05 was considered significant.

## Results

### Berberine treatment reduces triacylglycerol accumulation in the heart, skeletal muscle and liver of GDM exposed offspring

As previously reported^12^, BBR treatment attenuated body weight gain in HF fed offspring from both lean and GDM dams (Table 2). Next, we examined plasma lipids levels to determine how BBR affected lipid homeostasis in GDM offspring. Plasma levels of NEFA did not differ between lean and GDM offspring fed a LF diet. Plasma levels of NEFA were elevated in HF fed offspring from lean dams and this was reduced by treatment with BBR. In addition, BBR treatment reduced plasma levels of TG in lean offspring fed the HF diet. Plasma levels of ketones were elevated in GDM offspring compared to lean offspring and BBR treatment significantly reduced plasma ketone levels in the HF fed GDM offspring. Thus, BBR treatment attenuated the HF diet induced increase in plasma lipid levels in lean offspring but not GDM offspring.

**Table 1:** PCR primers.

Gene	Forward primer	Reverse primer
TBP	ACCCTTCACCAATGACTCCTATG	TGACTGCAGCAAATCGCTTGG
TFIIB	TGGAGATTTGTCCACCATGA	GAATTGCCAAACTCATCAAAACT
TAZ	GACCCTCATCTCTGGGGGAT	CAGCTCCTTGGTGAAGCAGA
PTPMT	GCAACACCTCGAAGGAATGG	GAGATTGGCCAAGGTTGGGA
PGS	ACACAGGTTCCAGTGGATCAG	TTTATCTGCCCCTTCATGAGC
PGC1	ATACCGCAAAGAGCACGAGAAG	CTCAAGAGCAGCGAAAGCGTCACAG
SIRT1	GAAACCTTTGCCTCATCTACA	CACCTAGCCTATGACACAACT
CPT1	ATGGCGGAAGCACACCAGGC	CGCCCAGACTCCGGTGGAGA
SREBP1 CD36	ATCCAAGGGCATCTGAGAACTC TGGCTAAATGAGACTGGGACC	GGCTATTCCGTGAACATCTCCTA ACATCACCACTCCAATCCCAAG
FABP1	CTGGCACTGGTGGACGCCAG	TTGCCTGGGGCTTGTGCCAG
FABP4	TGGAAGCTTGTCtCCAGTGA	AATCCCCATTTACGCTGATG
NDUFS3 SDHD	CTGACTTGACGGCAGTGGAT GATCCCTGCTTGGGTACTTGA	CATACCAATTGGCCGCGATG AAGTAGCAAAGCCCAGCAA
COX5β	CGTCCATCAGCAACAAGAGA	AGATAACACAGGGGCTCAGT
ATP5i	CCCCTGCTGAAATCCCTACA	TAAAACCACATCCGCACCTC

*TBP* tata box binding protein, *TFIIB* transcription factor IIB, *TAZ* tafazzin, *PGS* phosphatidylglycerolphosphate synthase, *PTPMT* protein tyrosine phosphatase mitochondrial 1, *PGC1α* peroxisome proliferator-activated receptor gamma coactivator 1α, *SIRT1* sirtuin 1, *CPT1β* carnitine palmitoyltransferase 1, *SREBP1* sterol regulatory element-binding protein 1, *ACOX1* acyl-CoA oxidase, *FABP* fatty acid binding protein, *NDUF* NADH dehydrogenase oxidoreductase core subunit S3, *SDHD* succinate dehydrogenase complex subunit D, *COX5β* cytochrome c oxidase subunit 5β, *ATP5i* ATP synthase subunit 5i.

**Figure 1:**
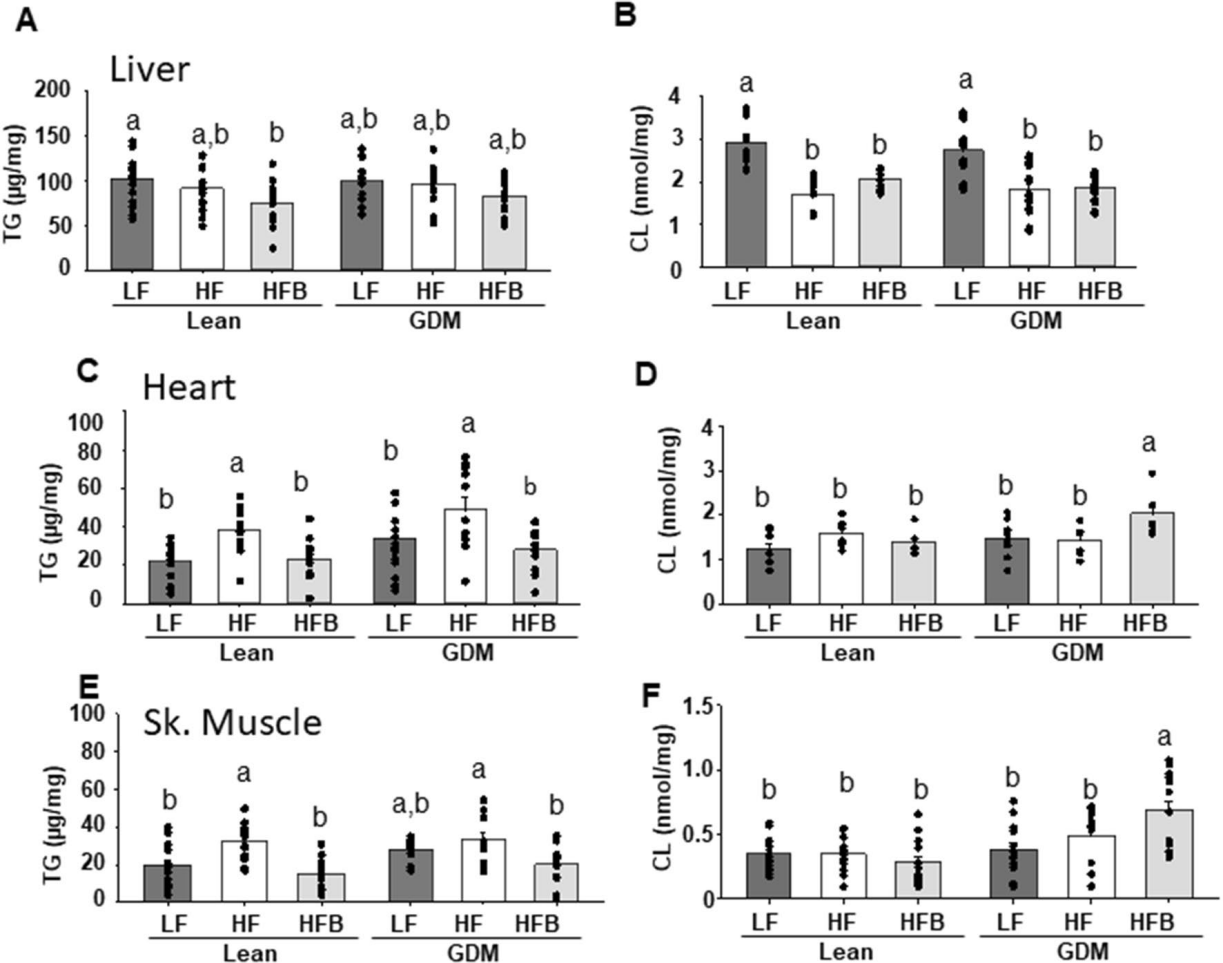
Triacylglycerol (TG) and total cardiolipin (CL) levels in liver, heart and skeletal muscle. All measurements were obtained following 12 weeks of the indicated diet (LF, HF, HFB). Values are means ± SEMs (n = 5–15). Means without a common letter differ, p < 0.05. *GDM* gestational diabetes mellitus.

We determined that BBR broadly reduced TG accumulation in the liver, heart and skeletal muscle (Fig. 1A,C,E). Specifically, an elevation in TG (~ 50%, p < 0.05) which occurred in the heart of both the HF fed Lean and GDM exposed offspring, was normalized in both groups of HFB offspring (Fig. 1C). A similar BBR mediated reduction in TG accumulation was observed in skeletal muscle (Fig. 1E). In the livers of lean offspring, BBR induced a more modest, but significant (~ 25%, p < 0.05) reduction in TG (Fig. 1A).

While significant modification of hepatic gene expression occurred in response to HF diet, including increased fatty acid (FA) uptake genes (*Fatbp1*, *Fatbp4*) and reduced fatty acid oxidation (FAO) genes (*Pgc1a*, *Sirt1*), these levels were not further augmented by GDM exposure (Table 3). When BBR was supplemented in the diet, the significant reduction in hepatic TG levels was limited to the lean gestational group (HFB Lean, Fig. 1A). This result was mirrored by significant alterations in hepatic gene expression (increased FAO and reduced FA uptake) in the HFB Lean offspring (Table 3)^19,20^. In the HF fed offspring, GDM-exposure promoted a robust increase in the gene expression of several mitochondrial respiratory complexes (*Nduf*, *Cox5b*). The increased mitochondrial respiratory complex gene expression was attenuated in HFB mice. The elevation in the gene expression of respiratory complexes may be a compensatory mechanism in the liver of GDM HF fed offspring to maintain mitochondrial function.

**Table 2:** Characteristics of offspring. All measurements were obtained following 12-weeks of the indicated diet (LF, HF, HFB).

	LEAN	LEAN	LEAN	GDM	GDM	GDM
	LF	HF	HFB	LF	HF	HFB
Body weight (g)	26.3 ± 0.529^c^	27.1 ± 0.808^c^	22.5 ± 0.431^e^	29.1 ± 0.522^b^	31.3 ± 0.895^a^	24.7 ± 0.500^d^
NEFA (µmol/L)	402 ± 35.9^a,b^	455 ± 31.7^b^	298 ± 34.7^a^	393 ± 29.3^a,b^	364 ± 38.8^a,b^	338 ± 29.3^a,b^
TG (mg/dL)	20.0 ± 1.33^a,b^	28.0 ± 3.22^a^	17.9 ± 2.44^b^	22.6 ± 2.48^a^	29.6 ± 3.55^a^	29.7 ± 4.01^a^
Ketones (mmol/L)	0.440 ± 0.0961^b^	0.514 ± 0.0751^b^	0.689 ± 0.0884^a,b^	0.822 ± 0.0961^a^	0.770 ± 0.101^a^	0.524 ± 0.0651^b^
ALT (IU/L)	35.8 ± 7.00^b^	28.9 ± 5.92^b^	30.9 ± 6.39^b^	40.5 ± 4.72^a,b^	46.7 ± 5.54^a^	37.7 ± 4.72^a,b^

Values are means ± SEMs (n = 5–15), means without a common letter differ, p < 0.05.

*NEFA* non-esterified fatty acids, *ALT* alanine aminotransferase.

We next assessed hepatic cardiolipin (CL) content since it is well established that this phospholipid is required for normal mitochondrial bioenergetics function^13^. HF diet significantly reduced hepatic CL in both lean and GDM offspring (Fig. 1B) (Table 4). Consistent with the lack of BBR mediated protection against mitochondrial dysfunction in the liver^12^, BBR treatment did not restore hepatic CL content (Fig. 1B). Minimal alterations in other phospholipids were observed by BBR treatment of GDM offspring (Table 4). In addition, plasma levels of ALT were not appreciably altered with BBR treatment of HF diet fed GDM offspring, suggesting normal liver function (Table 2). Together these data indicate that BBR treatment of GDM offspring had a minimal effect on hepatic mitochondrial parameters.

**Table 3:** PCR analysis of liver.

Gene	LEAN	LEAN	LEAN	GDM	GDM	GDM
	LF	HF	HFB	LF	HF	HFB
**CL synthesis**						
TAZ	1.00 ± 0.11^a,b^	0.84 ± 0.12^b^	1*.*21 ± 0.20^a.b^	1.00 ± 0.18^a,b^	1.67 ± 0.61^a^	0*.*82 ± 0.10^b^
PGS	1.00 ± 0.18	1.17 ± 0.35	0.97 ± 0.26	1.32 ± 0.34	1*.*34 ± 0.26	0.97 ± 0.26
PTPMTI	1.00 ± 0.14	0.82 ± 0.16	0.70 ± 0.18	1.19 ± 0.27	1.11 ± 17	0.85 ± 0.13
**FAO**						
PGC1α	1.00 ± 0.14^a,b^	0.67 ± 0.11^b^	1.29 ± 0.11^a^	1.31 ± 0.25^a^	0.74 ± 0.11^b^	0.57 ± 0.04^b^
SIRT1	1.00 ± 0.12^a^	0.60 ± 0.10^b^	0.83 ± 0.08^a,b^	0.90 ± 0.10^a,b^	0.66 ± 0.15^b^	0.77 ± 0.16^a,b^
CPT1β	1.00 ± 0.27^b^	1.13 ± 0.24^b^	3.32 ± 1.09^a^	0.40 ± 0.11^c^	0.88 ± 0.14^b^	0.73 ± 0.24^b^
SREBP1	1.00 ± 0.28	1.80 ± 0.37	1.11 ± 0.18	1*.*07 ± 0.22	2*.*10 ± 0.68	1.24 ± 0.11
ACOX1	1.00 ± 0.06^a^	0.84 ± 0.11^a^	0.39 ± 0.12^b^	0.84 ± 0.35^a^	0.55 ± 0.09^a,b^	0.57 ± 0.18^a,b^
**FA uptake**						
CD36	1.00 ± 0.10^a^	0.59 ± 0.08^b^	0.53 ± 0.11^b^	0.97 ± 0.14^a^	0.98 ± 0.19^a^	0.73 ± 0.09^a,b^
FABP1	1.00 ± 0.14^a,b^	1.26 ± 0.21^a^	0.94 ± 0.12^b^	0.80 ± 0.06^b^	1.01 ± 0.09^a^	0.81 ± 0.03^b^
FABP4	1.00 ± 0.14^a,b^	1.62 ± 0.27^a^	1.20 ± 0.51^a,b^	0.49 ± 0.10^b^	2.03 ± 0*.*12^a^	0.87 ± 0.14^b^
**ETC**						
NDUFS3 (I)	1.00 ± 0.12^a,b^	1.10 ± 0.31^a,b^	1.25 ± 0.18^a^	0.55 ± 0.07^b^	1.51 ± 0.17^a^	0.78 ± 0.07^b^
SDHD (II)	1.00 ± 0.08^b^	1.08 ± 0.21^b^	2.82 ± 0.58^a^	1.25 ± 0.22^a,b^	1.96 ± 0.31^a^	1.44 ± 0.22^a,b^
COX5β (IV)	1.00 ± 0.25^b^	0.82 ± 0.36^b^	0.97 ± 0.27^b^	2.18 ± 0.50^a^	2.34 ± 0.65^a^	0.88 ± 0.43^b^
ATP5i (V)	1.00 ± 0.20	0.77 ± 0.09	0.86 ± 0.19	0.88 ± 0.23	0.98 ± 0.18	0.86 ± 0.19

mRNAs were measured in the liver of offspring following 12-weeks of the indicated diet (LF, HF, HFB). Values are means ± SEMs (n = 7), means without a common letter differ, p < 0.05.

*TAZ* tafazzin, *PGS* phosphatidylglycerolphosphate synthase, *PTPMT* protein tyrosine phosphatase mitochondrial 1, *PGC1α* peroxisome proliferator-activated receptor gamma coactivator 1α, *SIRT1* sirtuin 1, *CPT1β* carnitine palmitoyltransferase 1, *SREBP1* sterol regulatory element-binding protein 1, *ACOX1* acyl-CoA oxidase, *FABP1* fatty acid binding protein 1, *NDUF* NADH dehydrogenase oxidoreductase core subunit S3, *SDHD* succinate dehydrogenase complex subunit D, *COX5β* cytochrome c oxidase subunit 5β, *ATP5i* ATP synthase subunit 5i.

### Berberine treatment increases cardiolipin in the heart and skeletal muscle of GDM exposed offspring

In the heart and skeletal muscles, CL content was similar among the Lean and GDM offspring LF and HF-fed groups. Interestingly HFB did not affect CL levels in the Lean offspring, but increased CL (~ 42%, p < 0.05) in the GDM offspring (Fig. 1D,F). Interestingly, in the heart this was due to significant increases (~ 32%, p < 0.05) in the most abundant and functionally important CL species, tetralinoleoylcardiolipin (L_4_CL) (1448) (Table 5). A BBR-mediated increase (47%, p < 0.05) in mRNA expression of tafazzin, the major CL remodeling enzyme^13^, may account for this increase in cardiac CL content in GDM HF offspring compared to lean HF offspring (Table 6). In addition, further PCR analysis of the heart indicated elevations in expression of enzymes involved in FA uptake, FAO (*PGC1α, CD36, FABP1, CTP1β*) and ETC subunits (*NDUF(I), SDHD(II)*) of the GDM exposed groups compared to lean animals (Table 6). This additionally would account for the previous observation of a BBR mediated improvement of cardiac mitochondrial function in HF diet fed GDM offspring^12^. Minimal alteration in other cardiac phospholipids was observed by BBR treatment of GDM offspring. Together, these results indicate that the BBR mediated protection from cardiac dysfunction in GDM exposed offspring, in part, involves its effects on improvements in mitochondrial function through increased CL synthesis.

**Table 4:** Quantitation of phospholipids and CL molecular species in the liver. Quantitation of phospholipids and molecular species of CL from the liver of offspring following 12-weeks of the indicated diet (LF, HF, HFB).

Phospholipid species	LEAN	LEAN	LEAN	GDM	GDM	GDM
	LF (nmol/mg)	HF (nmol/mg)	HFB (nmol/mg)	LF (nmol/mg)	HF (nmol/mg)	HFB (nmol/mg)
**CL species**						
1422	0.337 ± 0.027^a^	0.100 ± 0.012^c^	0.083 ± 0.012^c^	0.269 ± 0.021^b^	0.119 ± 0.018^c^	0.075 ± 0.005^c^
1424	0.753 ± 0.050^a^	0.153 ± 0.059^b^	0*.*117 ± 0.016^b^	0.683 ± 0.047^a^	0.207 ± 0.035^b^	0.124 ± 0.010^b^
1448	0.609 ± 0.033^b^	0.720 ± 0.059^b^	0*.*959 ± 0.042^a^	0*.*554 ± 0.065^b^	0.696 ± 0.069^a,b^	0.814 ± 0.047^a^
1450	0.792 ± 0.063^a^	0*.*480 ± 0.044^b^	0.604 ± 0.024^b^	0.725 ± 0.067^a^	0.508 ± 0.054^b^	0.546 ± 0.027^b^
1472	0.118 ± 0.013^a^	0.075 ± 0.006^b^	0.090 ± 0.003^a,b^	0*.*129 ± 0.011^a^	0.086 ± 0.008^b^	0.087 ± 0.004^b^
1474	0.238 ± 0.021^a^	0*.*087 ± 0.089^b^	0.117 ± 0.011^b^	0.221 ± 0.019^a^	0.128 ± 0.021^b^	0.109 ± 0.006^b^
1496	0.058 ± 0.005	0.050 ± 0*.*004	0.060 ± 0*.*005	0.059 ± 0.005	0.052 ± 0*.*006	0.049 ± 0*.*003
1498	0.064 ± 0.014^a^	0.017 ± 0*.*002^b^	0.030 ± 0*.*003^b^	0.050 ± 0*.*004^a^	0.023 ± 0*.*005^b^	0.028 ± 0*.*001^b^
**PC**	59.6 ± 2.13^a^	49.6 ± 2.06^b^	51.1 ± 1.51^b^	57.2 ± 2.69^a^	51.8 ± 3.84^c^	52.0 ± 2.16^c^
**PE**	33.6 ± 1.26^a^	21.9 ± 1.06^c^	28.1 ± 1.24^b^	33.0 ± 1.47^a^	24.9 ± 2.43^c^	23.8 ± 1.30^c^
**PS**	4.1 ± 0.17	3.1 ± 0.29	4.1 ± 0.45	3.7 ± 0.21	3.7 ± 0.42	3.5 ± 0.19
**PI**	11.7 ± 0.67^a^	9.9 ± 0.68^a,b^	13.4 ± 1.42^a^	11.4 ± 1.30^a^	9.1 ± 1.33^a,b^	6.6 ± 0.78^b^

Values are means ± SEMs (n = 7–10), means without a common letter differ, p < 0.05.

**Table 5:** PCR analysis of the heart. mRNAs were measured from the heart of offspring following 12-weeks of the indicated diet (LF, HF, HFB).

Gene	LEAN	LEAN	LEAN	GDM	GDM	GDM
	LF	HF	HFB	LF	HF	HFB
**CL synthesis**						
TAZ	1.00 ± 0.09^b^	1.38 ± 0.11^a^	0.87 ± 0.05^b^	1.16 ± 0.08^a,b^	1.19 ± 0.10^a^	1*.*28 ± 0.05^a^
PGS	1.00 ± 0.21^b^	1.38 ± 0.05^a^	0.90 ± 0.10^b^	1.13 ± 0.13^a,b^	1*.*40 ± 0.15^a^	1*.*06 ± 0.08^b^
PTPMTI	1.00 ± 0.10	1.00 ± 0.09	0.92 ± 0.04	1.00 ± 0.22	1.15 ± 16	1.10 ± 0.24
**FAO**						
PGC1α	1.00 ± 0.14^b^	1.12 ± 0.09^b^	1.02 ± 0.12^b^	1.79 ± 0.21^a^	1*.*62 ± 0.18^a^	1.46 ± 0.15^a^
SIRT1	1.00 ± 0.15	0.91 ± 0.07	0.79 ± 0.04	1.05 ± 0.22	0.82 ± 0.06	1.14 ± 0.10
CPT1β	1.00 ± 0.16^a,b^	1.16 ± 0.19^a,b^	0.83 ± 0.15^b^	1.06 ± 0.22^a,b^	1.51 ± 0.17^a^	1.52 ± 0.22^a^
ACOX1	1.00 ± 0.23^a,b^	0.37 ± 0.10^b^	1.45 ± 0.28^a^	1.10 ± 0.32^a,b^	1.13 ± 0.28^a,b^	1*.*40 ± 0.31^a^
**FA uptake**						
CD36	1.00 ± 0.10^a^	0.50 ± 0.06^b^	0.54 ± 0.09^b^	1.37 ± 0.14^a^	0.65 ± 0.07^b^	1.02 ± 0.14^a^
FABP1	1.00 ± 0.14^a^	0.52 ± 0.05^b^	0.50 ± 0.08^b^	0.63 ± 0.10^b^	0.64 ± 0*.*04^b^	1.00 ± 0.19^a^
**ETC**						
NDUF (I)	1.00 ± 0.10^a^	0.67 ± 0.04^b^	0.70 ± 0.09^b^	1.15 ± 0.18^a^	0.84 ± 0.08^a,b^	1.01 ± 0.17^a^
SDHD (II)	1.00 ± 0.20^a^	0.78 ± 0.06^a,b^	0.54 ± 0.09^b^	0.90 ± 0.24^a,b^	0.61 ± 0*.*09^b^	1.24 ± 0.18^a^
COX5β (IV)	1.00 ± 0.08	1.27 ± 0.06	1.02 ± 0.10	1.20 ± 0.14	1.30 ± 0.15	1.26 ± 0.16
ATP5i (V)	1.00 ± 0.12	1.11 ± 0.09	0.96 ± 0.13	1.05 ± 0.22	1.16 ± 0.12	1.15 ± 0.27

Values are means ± SEMs (n = 7), means without a common letter differ, p < 0.05.

*TAZ* tafazzin, *PGS* phosphatidylglycerolphosphate synthase, *PTPMT* protein tyrosine phosphatase mitochondrial 1, *PGC1α* peroxisome proliferator-activated receptor gamma coactivator 1α, *SIRT1* sirtuin 1, *CPT1β* carnitine palmitoyltransferase 1, *SREBP1* sterol regulatory element-binding protein 1, *ACOX1* acyl-CoA oxidase, *FABP* fatty acid binding protein, *NDUF* NADH dehydrogenase oxidoreductase core subunit S3, *SDHD* succinate dehydrogenase complex subunit D, *COX5β* cytochrome c oxidase subunit 5β, *ATP5i* ATP synthase subunit 5i.

**Table 6:** Quantitation of phospholipids and CL molecular species in the heart.

Phospholipid species	LEAN	LEAN	LEAN	GDM	GDM	GDM
	LF (nmol/mg)	HF (nmol/mg)	HFB (nmol/mg)	LF (nmol/mg)	HF (nmol/mg)	HFB (nmol/mg)
**CL species**						
1422	0.098 ± 0.010^a^	0.036 ± 0.001^b^	0.028 ± 0.004^b^	0.115 ± 0.014^a^	0.046 ± 0.008^b^	0.030 ± 0.003^b^
1424	0.055 ± 0.008^a^	0.020 ± 0.004^b^	0.023 ± 0.003^b^	0.057 ± 0.010^a^	0.023 ± 0.005^b^	0.030 ± 0.005^b^
1448	0.569 ± 0.077^b^	0.901 ± 0.059^a^	0.718 ± 0.073^b^	0.639 ± 0.081^b^	0.715 ± 0.07^b^	0.943 ± 0.072^a^
1450	0.180 ± 0.022^b^	0.222 ± 0.016^a,b^	0.232 ± 0.026^a^	0.232 ± 0.018^a,b^	0.249 ± 0.037^a^	0.283 ± 0.03^a^
1472	0.086 ± 0.03	0.100 ± 0.015	0.119 ± 0.021	0*.*115 ± 0.016	0.113 ± 0.012	0*.*151 ± 0.011
1474	0*.*050 ± 0.005	0*.*064 ± 0.007	0.063 ± 0.006	0.052 ± 0.007	0.065 ± 0.014	0.082 ± 0.011
1496	0.153 ± 0.011^b^	0.205 ± 0.024^a,b^	0.233 ± 0.018^a^	0.201 ± 0.030^a,b^	0.228 ± 0.035^a^	0.287 ± 0.025^a^
1498	0.044 ± 0.007^b^	0.044 ± 0.007^b^	0.086 ± 0.020^a^	0.061 ± 0.006^b^	0.057 ± 0.01^b^	0.107 ± 0.023^a^
**PC**	44.06 ± 1.88	48.91 ± 2.34	40.68 ± 2.00	43.51 ± 2.67	51.40 ± 3.74	45.70 ± 3.09
**PE**	24.30 ± 1.44	27.61 ± 2.18	22.69 ± 1.22	23.57 ± 2.00	28.81 ± 2.63	25.83 ± 1.87
**PS**	5.92 ± 0.36^a^	6.44 ± 0.54^a^	6.75 ± 0.33^a^	6.12 ± 0.45^a^	5.18 ± 0.57^a,b^	3.60 ± 0.67^b^
**PI**	4.12 ± 0.33^b^	5.57 ± 0.73^a,b^	5.46 ± 0.55^a,b^	6.24 ± 0.63^a^	6.17 ± 0.46a	4.45 ± 0.25^b^

Quantitation of phospholipids and molecular species of CL from the heart of offspring following 12-weeks of the indicated diet (LF, HF, HFB). Values are means ± SEMs (n = 8), means without a common letter differ, p < 0.05.

## Discussion

The major findings of this study are, (1) supplemental BBR in a HF diet attenuates tissue TG accumulation in offspring from lean and GDM dams, (2) The cardiometabolic protective effects of BBR in GDM offspring are minimally impacted by alterations in liver mitochondrial metabolism, (3) BBR treatment increased total CL in heart and skeletal muscle and L_4_CL levels in the heart of GDM offspring, (4) The increase in cardiac L_4_CL was likely due to an increase in expression of the CL remodeling enzyme tafazzin, and (5) Elevation in expression of cardiac enzymes involved in FA uptake, FAO and ETC subunits coupled with the increase in L_4_CL may account for the improved cardiac mitochondrial function in BBR treated GDM offspring.

Previously we demonstrated that that supplemental BBR in a high fat diet reduced adiposity and improved cardiac mitochondrial dysfunction in offspring of mouse dams with GDM fed a HF diet^12^. However, the mechanism for the improvement in cardiac function in these GDM offspring was unknown. Since abnormal lipid accumulation is associated with the development of cardiac and mitochondrial dysfunction, and the liver, heart and skeletal muscle play major roles in handling lipids^21^, we assessed whether cardiac, skeletal muscle and liver lipid homeostasis was altered by BBR treatment in GDM offspring fed a HF diet. Consistent with the role of BBR as a lipid-lowering agent^11^, we determined that the cardiovascular protective effect of BBR was mirrored by reduced TG accumulation in the liver, heart and skeletal muscle.

There are numerous studies on the beneficial effects of BBR on the prevention of hepatic steatosis^19,20,22^. Since GDM has previously been shown to promote hepatic steatosis^14,15^ and BBR attenuated TG accumulation in liver (Fig. 1), we investigated whether BBR altered gene expression of hepatic bioenergetics enzymes. While significant modification of hepatic gene expression occurred in response to HF diet for both FA uptake and FAO, these levels were not further augmented by GDM exposure. As previously reported, BBR treatment did not significantly improve hepatic mitochondrial CI or CII mediated oxygen consumption rate for any respiratory state measured^12^.

Based on evidence that BBR protected against HF diet induced mitochondrial dysfunction in muscle^23^ and our previous observation that dietary BBR prevented the HF diet mediated reductions in cardiac state I and state III respiration and normalized spare capacity in GDM offspring^12^, we assessed whether these protective effects accompanied changes in CL, the signature phospholipid that regulates mitochondrial bioenergetics^13^. BBR was effective at elevating CL content in both the heart and skeletal muscle of the GDM exposed groups. Interestingly, in the heart this was due to an increase in L_4_CL, the most abundant and functionally important CL species^13^. PCR analysis indicated an increase in mRNA expression of tafazzin, a CL remodeling enzyme, which likely accounted for this increase in cardiac L_4_CL content. Studies in mice have indicated that tafazzin deficiency in heart accompanies reduced cardiac L_4_CL and mitochondrial bioenergetic function^24–27^. This is underscored by the X-linked genetic disease Barth Syndrome in which mutations in tafazzin result in reduced cardiac CL and L_4_CL levels in patients^28^.

Moreover, mRNA gene expression analysis demonstrated that increased expression of enzymes involved in FA uptake, FAO and ETC subunits in GDM HF exposed groups compared to lean animals could contribute to our previous observation of a BBR mediated improvement of cardiac function in HF diet fed GDM offspring^12^. Together, our results indicate that the BBR mediated protection from cardiac dysfunction in GDM HF exposed offspring involve its effects on improvements to mitochondrial function which are mediated, in part, through increased CL synthesis.
